# The Hidden Dangers: E-Cigarettes, Heated Tobacco, and Their Impact on Oxidative Stress and Atherosclerosis—A Systematic Review and Narrative Synthesis of the Evidence

**DOI:** 10.3390/antiox13111395

**Published:** 2024-11-15

**Authors:** Arianna Magna, Nausica Polisena, Ludovica Polisena, Chiara Bagnato, Elena Pacella, Roberto Carnevale, Cristina Nocella, Lorenzo Loffredo

**Affiliations:** 1 Department of Clinical Internal, Anesthesiological and Cardiovascular Sciences, Sapienza University of Rome, Viale del Policlinico 155, 00161 Rome, Italy; 2 Department of Sense Organs, Faculty of Medicine and Dentistry, Sapienza University of Rome, 00161 Rome, Italy; 3 Department of Medical-Surgical Sciences and Biotechnologies, Sapienza University of Rome, 04100 Latina, Italy; 4 IRCCS—Istituto di Ricovero e Cura a Carattere Scientifico Neuromed, 86077 Pozzilli, Italy

**Keywords:** smoking, oxidative stress, heated tobacco products, cigarettes, electronic cigarettes, NADPH oxidase, endothelial dysfunction, platelets, atherosclerosis, thrombosis

## Abstract

Electronic cigarettes and heated tobacco products have seen significant growth in sales and usage in recent years. Initially promoted as potentially less harmful alternatives to traditional tobacco, recent scientific evidence has raised serious concerns about the risks they pose, particularly in relation to atherosclerosis. While atherosclerosis has long been associated with conventional tobacco smoking, emerging research suggests that electronic cigarettes and heated tobacco may also contribute to the development of this condition and related cardiovascular complications. In a narrative review, we examined the potential effects of heated tobacco products and electronic cigarettes on oxidative stress and atherosclerosis. Several studies have shown that e-cigarettes and heated tobacco increase oxidative stress through the activation of enzymes such as NADPH oxidase. One of the primary effects of these products is their pro-thrombotic and pro-atherosclerotic impact on endothelial cells and platelets, which promotes inflammatory processes within the arteries. Furthermore, the chemicals found in electronic cigarette liquids may exacerbate inflammation and cause endothelial dysfunction. Furthermore, through a systematic review, we analyzed the effects of chronic exposure to electronic and heated tobacco cigarettes on endothelial function, as assessed by brachial flow-mediated dilation (FMD). Although electronic cigarettes and heated tobacco cigarettes are often perceived as safer alternatives to traditional smoking, they could still present risks to cardiovascular health. It is essential to raise public awareness about the potential dangers associated with these products and implement protective measures, particularly for young people.

## 1. Introduction

In recent years, the debate surrounding the safety of electronic cigarettes and heated tobacco products has taken on an increasingly prominent role in the scientific literature [1]. Initially, these alternatives to conventional tobacco were perceived as a potential way out for smokers [1]. However, recent scientific evidence has begun to raise significant concerns about the risks associated with the use of these products, particularly concerning atherosclerosis [1].

Atherosclerosis is a pathological condition characterized by the inflammation of the arteries and the accumulation of lipids and macrophages within their walls. Classic cardiovascular risk factors, primarily cigarette smoking and high cholesterol, but also diabetes, hypertension, and obesity, can promote the onset of this pathological process from an early age. Over time, atherosclerosis can lead to serious cardiovascular complications such as myocardial infarction, stroke, and sudden death [2].

While atherosclerosis has traditionally been associated with conventional tobacco smoking, recent research has suggested that electronic cigarettes and heated tobacco products may also contribute to the development of this condition [3].

One of the main effects of electronic cigarettes and heated tobacco products is their pro-thrombotic and pro-atherosclerotic impact on endothelial cells and platelets, promoting inflammatory processes in the arteries. Additionally, some studies suggest that the chemical compounds present in the liquids of electronic cigarettes may contribute to inflammation and endothelial dysfunction [3,4,5]. Given the increasingly widespread use of these products, particularly among younger people, the purpose of this narrative review is to analyze the potential effects of electronic cigarettes and heated tobacco products on atherosclerosis and the consequent cardiovascular risk. Furthermore, we executed a systematic review to analyze the effect of chronic exposure to heated tobacco products and electronic cigarettes on endothelial function evaluated by brachial flow-mediated dilation (FMD).

## 2. Epidemiology

Tobacco use is one of the leading risk factors globally, particularly for cardiovascular diseases [6]. It is estimated that over 7 million people die each year due to the harmful effects of tobacco smoke, which is equivalent to approximately one death every six seconds [6]. Of these deaths, about 5–7 million are directly attributable to smokers, while over 1 million are caused by exposure to secondhand smoke. Worldwide, more than 40% of children have at least one parent who smokes [6].

Electronic cigarettes and heated tobacco products were recently introduced into the market as a less harmful alternative to traditional cigarettes generating an aerosol without the exothermic combustion typical of traditional cigarettes. Anyway, in recent years, there has been a large increase in the widespread use of these new-generation tobacco products, especially among young people aged 18 to 34 [6].

According to WHO estimates, about 6% of adolescents aged 13 to 15 are smokers, with a higher prevalence in Europe (7.8%) compared to the rest of the world, and one of the highest rates is in Italy (19.8%) compared to the other European countries [6,7]. Regarding new-generation products, according to the Global Youth Tobacco Survey (GYTS) data, the prevalence of use of e-cigs among young adults is even higher (15.5% in Europe and 17.5% in Italy), with a high prevalence of dual users (6.7% in Europe and 10% in Italy) [6,7]. As for exposure to secondhand smoke, even if there was a significant decrease in Europe in 2018 (from 53.7% to 38%), exposure to secondhand smoke is still 49.7% at home and 45% in public places among adolescents aged 13 to 15, which is still a very high prevalence given the well-known health risk related to smoke [8].

These epidemiological data underscore the urgent need to implement targeted strategies, especially among young people, to curb the increasing use of cigarettes and prevent future health complications.

## 3. Electronic Cigarette

Introduced in Europe in 2006, electronic cigarettes are often considered a less harmful alternative to traditional cigarette smoking, as they avoid the combustion of tobacco [9].

The electronic cigarette (e-cig, Electronic Nicotine Delivery System) consists of four main parts: a “mouthpiece”, which is placed on the lips and allows the user to inhale the vapor; a “cartridge”, which contains the e-liquid, composed of nicotine (available in various concentrations or nicotine-free), propylene glycol, glycerol, flavorings, and other additives; an “atomizer”, which heats the e-liquid until it evaporates completely; and a “rechargeable battery”, which can be activated either by pressing a button or automatically by inhaling [9].

However, numerous studies have shown that the vapor produced by these electronic devices, while associated with fewer side effects, can cause respiratory and cardiovascular alterations both in the short and long term [9]. The aerosol produced by the evaporation of the e-liquid contains the substances in the liquid, such as propylene glycol, nicotine, and flavorings. However, it also contains newly formed substances such as propylene oxide, acrolein, acetaldehyde, formaldehyde, acetamide, metals (including silver, copper, and nickel), and silicate particles, all of which have been proven to have harmful effects on humans [9]. A previous human study has demonstrated significant increases in the urinary metabolites of acrolein, propylene oxide, acrylamide, and acrylonitrile in e-cigarette smokers [10]. Some e-liquid flavorings also appear to have harmful effects on health. For example, cinnamaldehyde, 2-methoxycinnamaldehyde, O-vanillin, and pentanedione have been shown to have cytotoxic effects on the respiratory system [11]. Additionally, the composition of the atomizer seems to influence the toxic effects of these devices: steel atomizers do not appear to cause acute respiratory difficulties in murine models, unlike those made from nickel-chromium alloys [9].

## 4. Heated Tobacco Products

In recent years, a new smoking technology called the heat-not-burn cigarette (HNBC) has been introduced to the market. These non-combustible tobacco devices consist of three elements: the heatstick, the holder, and the charger. The heatstick is composed of small sheets of tobacco containing 70% tobacco, water, glycine (a humectant that promotes aerosol formation), flavorings, and a filter necessary for cooling the aerosol. When in use, the heatstick is inserted into the holder, which contains an electronic blade that heats the tobacco mixture to a temperature of about 350 °C. This process dries the tobacco, evaporates volatile products like nicotine, and causes the thermochemical decomposition of the tobacco (low-temperature roasting/pyrolysis) without generating solid particles. The heating process is regulated by the holder and automatically stops after a set time or number of puffs to prevent the pyrolysis process.

The lower temperatures used by these devices allow for the release of nicotine and other volatile compounds without relying on the combustion process typical of traditional cigarettes. In traditional cigarette combustion, there are two zones: an exothermic combustion zone, generating heat between 700 and 900 °C, and a pyrolysis zone, which is low in oxygen and reaches temperatures of 200–600 °C. It is in this pyrolysis zone that most of the combustion products are created through endothermic mechanisms.

Heated tobacco products were introduced to the market as a less harmful alternative to traditional cigarettes because they generate an aerosol without the exothermic combustion typical of traditional cigarettes, avoiding the pyrolysis processes responsible for producing most of the harmful and potentially dangerous components of tobacco [12,13,14]. However, studies conducted after the market introduction of these products have shown that the temperatures at which HNBC generates aerosol (up to 320–350 °C) are sufficient to initiate the same endothermic pyrolytic processes found in traditional cigarettes [15]. The aerosol from these new products has been found to contain multiple substances harmful to humans, such as acrolein, aldehydes, metals, and pyrolysis-derived products like tobacco-specific nitrosamines (TSNAs) and polycyclic aromatic hydrocarbons (PAHs) [14]. This should prompt a reconsideration of the concept of “smoke-free aerosol” associated with the use of these devices. Future studies will need to evaluate the potential health damage from chronic exposure to these substances in HNBC users.

## 5. Effects of Smoking on the Endothelium and Oxidative Stress

The link between smoking and cardiovascular disease is well established, though the underlying pathophysiological mechanisms are complex and not yet fully understood. One proposed mechanism involves endothelial dysfunction. This dysfunction is a critical indicator of cardiovascular damage and is considered an early predictor of cardiovascular events and prognosis in smokers [16].

Endothelial dysfunction can be non-invasively assessed in humans through flow-mediated dilation (FMD), an ultrasound-based method [17]. After a transient ischemic stimulus, such as the inflation of a sphygmomanometer cuff above systolic levels on the forearm, blood flow in the brachial artery increases in response to acute ischemia [17]. Healthy endothelial cells release factors, including nitric oxide (NO), which mediate smooth muscle relaxation, leading to acute vasodilation measurable by ultrasound [18]. In the case of endothelial dysfunction, reduced NO bioavailability contributes to diminished vasodilation and thus lower FMD values [18].

Functional endothelial cells are crucial for regulating vascular tone, inflammation, and platelet aggregation. The vasodilatory action of the molecules released by the endothelium has anti-atherosclerotic and anti-aggregating effects, fundamental mechanisms in preventing atherosclerotic disease. Reduced FMD values are associated with an increased risk of cardiovascular events [16].

**Figure 1:**
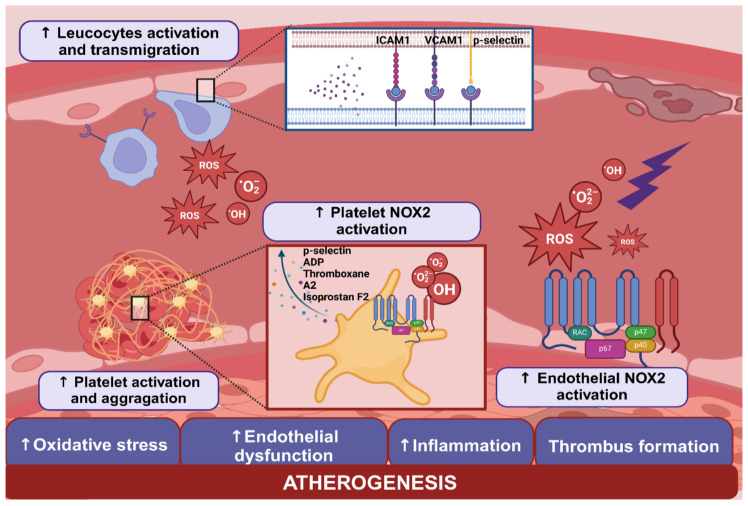
Effects of smoking on endothelial function. Smoking leads to an increased concentration of circulating ROS (reactive oxygen species) due to both the inhaled fraction absorbed in the lungs and endogenous production. One of the main endogenous sources of ROS is nicotinamide adenine dinucleotide phosphate oxidase (NADPH oxidase, NOX), a multimeric transmembrane protein present in both endothelial cells and platelets. NOX catalyzes the transfer of electrons from cytoplasmic NADPH to molecular oxygen, resulting in the formation of O_2_^−^ and H_2_O_2_, which leads to a reduction in NO (nitric oxide) and an increase in oxidative stress. Smoking also induces the transmigration of activated leukocytes by increasing the expression of adhesion molecules (ICAM-1, VCAM-1, and E-selectin) on endothelial cells. Furthermore, smoking promotes platelet activation, leading to the release of molecules such as P-selectin, ADP, thromboxane A2, and isoprostane F2, which amplify platelet activation and aggregation. These mechanisms cause endothelial damage and contribute to altering the endothelial phenotype from a physiological anti-thrombotic state to a pro-thrombotic and pro-inflammatory state, ultimately promoting the atherosclerotic process.

The pathophysiological mechanisms underlying smoking-induced endothelial dysfunction are intricate. Oxidative stress plays a key role, increasing the production of reactive oxygen species (ROS) and reducing nitric oxide (NO) bioavailability (Figure 1) [19,20].

The increased concentration of ROS is due to both the inhalation of smoke and endogenous production. One primary endogenous source of ROS is the enzyme NADPH oxidase (NOX), which catalyzes the transfer of electrons from cytoplasmic NADPH to molecular oxygen, resulting in the formation of superoxide anions that react with NO to form peroxynitrite, a highly unstable species that can generate further ROS [21,22]. Tobacco smoke and exposure to substances such as ketones and aldehydes, found in both traditional cigarettes and newer smoking products, can activate NOX, leading to increased endogenous ROS production [21,22]. Other mechanisms include increased xanthine oxidase activity and the breakdown and inactivation of endothelial nitric oxide synthase (eNOS) [23]. Smoking also induces ROS production by leukocytes and enhances leukocyte transmigration by increasing the expression of adhesion molecules (ICAM-1, VCAM-1, and E-selectin) and triggering NF-kB transcription, shifting the endothelial phenotype from an anti-thrombotic to a pro-thrombotic and pro-inflammatory state [24,25,26,27,28]. This review evaluates relevant studies from the available literature on the cardiovascular effects of acute (short-term) and chronic (long-term) exposure to e-cigarettes and heated tobacco products, as summarized in Table 1.

In vitro studies have evaluated the effects of traditional cigarette smoke and newer smoking products (e-cigarettes and HNBC) on endothelial function. Giebe et al. reported significant impairment in cell viability and the repair capacity of endothelial damage only with traditional cigarette extracts, with increased oxidative stress [29,30]. On the other hand, HNBC and e-cigarette extracts determined increased monocyte adhesion to endothelial cells and enhanced the expression and synthesis of pro-inflammatory genes and proteins, even if lower compared to traditional cigarettes [29,30]. Another in vitro study compared the effects of traditional cigarette smoke and HNBC smoke on endothelial function, assessing three HNBC devices with increasing tobacco heating temperatures (200 °C, 240 °C, and 300–350 °C) [29,30]. Although the cytotoxicity in endothelial cells exposed to traditional cigarette smoke was greater, devices with higher heating temperatures exhibited an increased particulate phase of smoke, characterized by a higher presence of cytotoxic carbonyl compounds, resulting in reduced mitochondrial metabolic activity and eNOS activity in endothelial cells [31]. Certain e-cigarette flavors also appear to be associated with increased endothelial dysfunction. A recent study on aortic endothelial cells showed that low concentrations of vanillin, menthol, cinnamaldehyde, eugenol, and acetylpyrazine induced increased ROS production, pro-inflammatory mediators, and reduced NO bioavailability [32].

There are limited data on chronic effects, but a recent study evaluated the in vitro effects of the chronic exposure of endothelial cells to e-cigarette smoke, revealing a degree of endothelial dysfunction comparable to that induced by chronic exposure to traditional cigarette smoke [33]. Cells exposed to e-cigarette smoke also showed increased microvascular permeability, elevated ICAM-1 levels (promoting leukocyte recruitment), and increased S100A8, a ligand that activates the RAGE receptor (Receptor for Advanced Glycation End Products), playing a role in transendothelial migration and cellular permeability [33].

The acute and chronic effects of new-generation tobacco smoke have also been evaluated in pre-clinical studies on murine models. Both single inhalation, short-term, and chronic exposure (up to 8 months) to these products showed alterations in endothelial function similar to those induced by traditional cigarette smoke [34,35,36]. This effect was confirmed when comparing different types of new-generation smoke products (e.g., HNBC and e-cigarettes), various flavors, and the presence or absence of nicotine. Acute exposure to a single session of any aerosol type resulted in FMD alterations in murine models comparable to those caused by traditional cigarette smoke [37].

Recent clinical studies have shown data consistent with in vitro and ex vivo findings. One study compared the acute effects of traditional tobacco smoke with e-cigarette smoke on endothelial function, oxidative stress, and vitamin E levels in smokers and non-smokers [38]. Both types of smoke were associated with elevated oxidative stress markers, reduced FMD, lower NO levels, and decreased vitamin E levels, with no statistically significant difference between traditional tobacco and e-cigarettes [38]. Another study measured endothelial progenitor cells and microvesicles as markers of endothelial damage in healthy young volunteers following short-term exposure to e-cigarette smoke [39]. In healthy subjects, the inhalation of 10 puffs of e-cigarette vapor led to an increase in endothelial progenitors similar to that induced by traditional cigarettes [39]. Another recent study evaluated e-cigarette-induced endothelial dysfunction by measuring nitric oxide levels in three different populations of young people (exclusive e-cigarette smokers for over a year, exclusive traditional tobacco smokers for over a year, and non-smokers). An interesting finding was that with equal cotinine levels, indicating similar smoking exposure, circulating nitric oxide levels were significantly lower in e-cigarette smokers compared to traditional cigarette smokers [40]. E-cigarette-induced endothelial dysfunction also appears to occur in the absence of nicotine. Caporale et al. demonstrated alterations in microvascular and macrovascular function parameters, assessed by the magnetic resonance imaging (MRI) of various vascular districts, after the inhalation of nicotine-free e-cigarettes in a population of healthy young non-smokers [41]. Consequently, endothelial dysfunction induced by e-cigarettes seems to occur even in the absence of nicotine, as it could be induced by the toxic metals contained and by the reduction in antioxidants such as vitamin E, for example [41].

There is still a limited number of clinical studies on the effects of HNBC smoking. A cross-sectional, randomized study compared the acute effects of HNBC use with those of electronic and traditional cigarettes, demonstrating that the single use of any product was associated with acute adverse effects on oxidative stress, platelet function, FMD, and blood pressure [42]. However, the effects were less pronounced with HNBC and electronic cigarettes compared to traditional cigarettes [42]. Another recent clinical study examined the risks of passive smoking from HNBC by evaluating oxidative stress parameters, endothelial dysfunction, and platelet activation in 78 children exposed to different types of secondhand smoke (HNBC, traditional cigarettes, and controls) [43]. Children exposed to secondhand smoke, both from HNBC and traditional cigarettes, showed significantly increased NOX2 activity, serum levels of H_2_O_2_, isoprostanes, and P-selectin, along with a significant reduction in FMD and NO compared to the controls, with no significant difference between the two types of smoke. Passive exposure to HNBC smoke could lead to increased oxidative stress, endothelial dysfunction, and platelet activation, with a higher risk of thrombosis in children [43].

There are still limited data on clinical studies on the chronic effects of electronic and HNBC use (Table 1). Mohammadi et al. [33] evaluated endothelial function in chronic smokers of electronic and traditional tobacco cigarettes (Table 1). Both groups of smokers exhibited alterations in the markers of inflammation, cell adhesion, and thrombosis. Compared to controls, the electronic cigarette smokers showed reduced FMD, lower serum NO levels, increased serum release of H_2_O_2_, and greater endothelial permeability. They also had an increased concentration of S100A8, a RAGE ligand, which was associated with increased microvascular permeability, a finding not observed in traditional tobacco smokers [33]. In another study by Loffredo et al. [4] the chronic effects of HNBC smoking were compared with those of traditional cigarette smoking and healthy controls. At comparable cotinine levels, the study found a significant reduction in NO and FMD among the smokers of both HNBC and traditional tobacco compared to non-smokers, with no significant difference between the HNBC and traditional tobacco smoker groups [4].

Unlike the studies mentioned above, Fotterman [44], Boakye [45] and Haptonstall [46] found no differences in flow-mediated dilation (FMD) between the users of electronic cigarettes, traditional tobacco, dual users, and non-smokers (Table 1).

**Figure 2:**
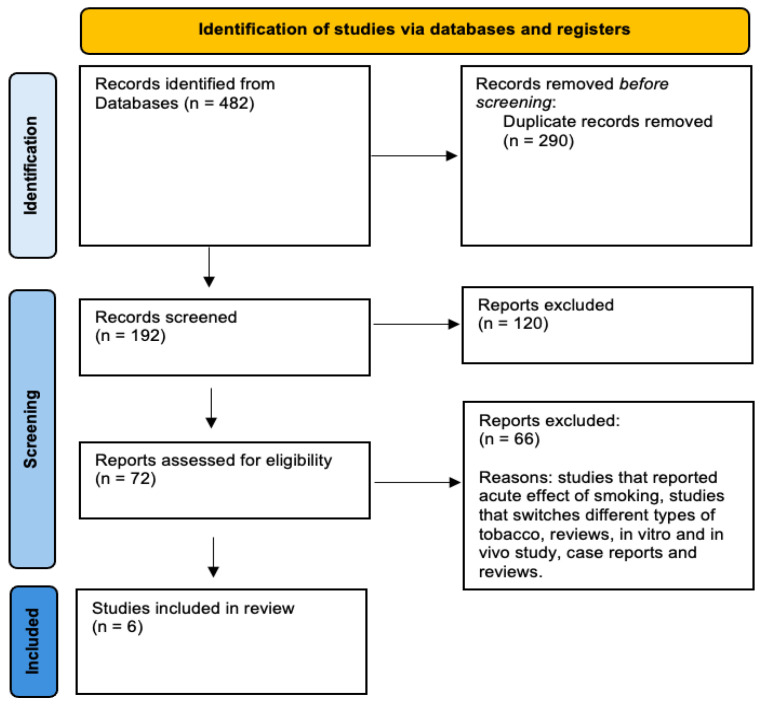
Flow diagram outlining the studies included in the systematic review to evaluate the effects of e-cigarettes and heated tobacco products on brachial FMD.

**Figure 3:**
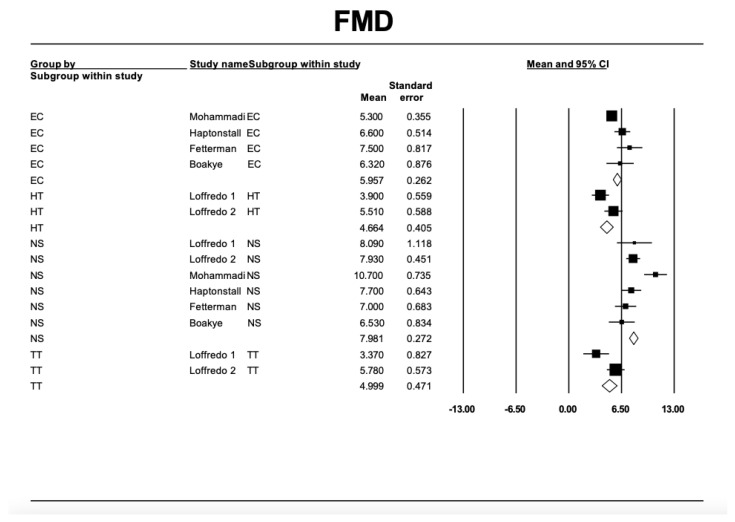
Forest plots illustrating the effects of exposure to e-cigarette (EC), heated tobacco (HT), or traditional tobacco (TT) smoke on brachial FMD (expressed as %). Related references: Mohammadi: [33], Loffredo 1: [43]; Loffredo 2: [4]: Fetterman: [44], Boakye: [45], Haptonstall: [46].

To better assess the chronic effects of exposure to heated tobacco or electronic cigarette smoke on endothelial dysfunction in the brachial artery, we conducted a systematic review (see the flow-diagram in Figure 2, Methods in Supplementary Data) [47]. The forest plots indicate that chronic exposure to the smoke from these alternative products is associated with reduced FMD values compared to non-smokers (Figure 3).

Recently, Ikonomidis [48] showed that switching from traditional cigarettes (tcig) to heated non-burn cigarettes (HNBC) for one month led to improvements in endothelial function, reduced oxidative stress, and decreased platelet activity. These interesting results merit further confirmation. Currently, all major lung and heart international societies do not recommend the use of electronic or heated tobacco products as a means to quit smoking.

Additional harm from alternative products to traditional cigarettes may also derive from the heavy metals they contain. Both e-cigarettes and heated tobacco products release toxic metals at higher concentrations than those found in conventional cigarette smoke [49]. Metals such as nickel and cadmium may contribute to oxidative stress, promoting DNA damage and activating oncogenes that support carcinogenesis [49]. Notably, cadmium and nickel may act as “metallo-estrogens” in breast cancer development; however, epidemiological studies indicate that cadmium, in particular, is more strongly associated with estrogen-like growth in breast malignancies [49].

**Table 1:** Studies on the acute and chronic cardiovascular effects of exposure to e-cigarettes and heated tobacco products.

Author	Type of Study	Exposure	Findings
Giebe S [29]	In vitro	Primary human endothelial cells exposed to aqueous smoke extracts (AqEs) of a heated tobacco product (HTP), an electronic cigarette (e-cig), a conventional cigarette (3R4F), and pure nicotine.	-3R4F stimulation, but not alternative smoking products, reduced endothelial cell viability and wound healing. -Aqueous extracts of different smoking products activated dose-dependent NRF2 antioxidant defense system in primary human endothelial cells. -Smoking leads to a statistically increased adhesion of monocytes to endothelial cells compared to controls (*p* < 0.05). -Stimulation with tobacco and nicotine products induces pro-inflammatory endothelial phenotype.
Giebe S [30]	In vitro	Monocytes exposed to aqueous smoke extracts (AqEs) of a heated tobacco product (HTP), an electronic cigarette (e-cig), a conventional cigarette (3R4F), and pure nicotine (nic).	-3R4F, but not next-generation tobacco, and nicotine products (NGPs) mediated cytotoxic effects on the cell viability of human monocytes. -Antioxidative signaling pathways are activated by the AqEs of tobacco and nicotine products. -Treatment with AqEs of different tobacco products regulate pro-inflammatory signaling pathways.
Horinouchi T [31]	In vitro	Human vascular endothelial cells exposed to the smoke of heated cigarette-derived smoke extract (hCSE) of three different cigarette heating devices and burned CSE (bCSE).	-hCSE/bCSE reduced the mitochondrial metabolic activity (MTS) in a statistically significant way (*p* < 0.01) in terms of delayed damage. The inhibitory effects were attenuated by removing the particulate phase from the mainstream smoke. -hCSE/bCSE reduced eNOS phosphorylation with different kinds of potency depending on the different kinds of devices.
Fetterman JL [32]	In vitro	Isolated endothelial cells from non-smoker and smoker participants who used nonmenthol- or menthol-flavored tobacco cigarettes; human aortic endothelial cells incubated with vanillin, menthol, cinnamaldehyde, eugenol, dimethylpyrazine, diacetyl, isoamyl acetate, eucalyptol, and acetylpyrazine.	-Endothelial cells collected from nonmenthol and menthol cigarette smokers had lower NO production in response to stimulation compared with cells from non-smokers (*p* = 0.003 non-smokers versus nonmenthol cigarette smokers; *p* = 0.012 non-smokers versus menthol cigarette smokers). -The impairment in stimulated nitric oxide production was similar between nonmenthol cigarette smokers and menthol cigarette smokers (*p* = 0.86). -Lower concentrations of selected flavors (vanillin, menthol, cinnamaldehyde, eugenol, and acetylpyridine) induced both inflammation and impaired A23187-stimulated nitric oxide production consistent with endothelial dysfunction.
Mohammadi L. [33]	In vitro and human	Chronic e-cigarette users, chronic cigarette smokers, and nonusers: FMD and cultured endothelial cells.	-FMD was reduced in both e-cigarette users and cigarette smokers relative to the nonusers (5.3 ± 2.3% and 6.5 ± 2.8% vs. 10.7 ± 5.2%, respectively, adjusted *p* = 0.0496 for smokers vs. nonusers, 0.0020 for ecig vs. nonusers). -Smokers and e-cigarette users had significantly lower NO production after stimulation than nonusers (adjusted *p* = 0.0496 for smokers vs. nonusers, 0.0093 for e-cigs vs. nonusers). -Clinically observed vascular dysfunction in smokers and e-cigarette users is paralleled by the inhibitory effects of serum on endothelial cell NO production, although the correlation was not observed on the per-participant level by Pearson analysis (r = 0.2). -S100A8, HMGB1, IFN-β, soluble ICAM-1, vWF, and myeloperoxidase (MPO) were unchanged in smokers but were substantially higher in e-cigarette users than in the other groups. -IL-1β (trend as *p* = 0.06), RAGE, and soluble PECAM-1 were unchanged in e-cigarette users and were elevated in cigarette smokers. -There was a significant increase in the level of RAGE ligands S100A8 and HMGB1 in serum from e-cigarette users compared to the other groups.
Nabazivadeh P [34]	Animal	Pre-exposure and post-exposure FMD of mice exposed to IQOS aerosol from single Heatsticks, mainstream smoke from single Marlboro Red cigarettes.	-FMD was reduced after 15 s exposures to IQOS aerosol (10.6 ± 2.9% pre-exposure vs. 4.5 ± 1.9% post-exposure, *p* = 0.0009) and cigarette smoke (10.6 ± 2.0% pre-exposure vs. 4.6 ± 1.3% post-exposure, *p* = 0.0004). FMD was not affected in the clean air control group (8.3 ± 1.9% vs. 8.8 ± 4.5%, *p* = 0.82). -FMD was impaired after 5 s exposures to IQOS aerosol and cigarette smoke (10.8 ± 1.0% pre-exposure vs. 3.8 ± 2.6% post-exposure, *p* = 0.0001; and 11.2 ± 2.6% pre-exposure vs. 4.2 ± 2.3% post-exposure, *p* = 0.0006, respectively). FMD was not affected in the air control group (9.5 ± 3.0% vs. 8.1 ± 1.8%, *p* = 0.85). -The percent FMD impairment was not significantly different in groups exposed for 5 s compared with 15 s (*p* = 0.27).
Kuntic M [35]	Animal	-Vascular (endothelial) mice function, oxidative stress, and inflammation after exposure to unflavored e-cigarette liquids with and without nicotine. -Evaluation of human endothelial cells	-E-cigarette vapor exposure reduced FMD (*p* = 0.017). -E-cigarette vapor exposure (with nicotine) for 1, 3, and 5 days caused endothelial dysfunction determined by acetylcholine-dependent relaxation in wild-type mice upon all exposure protocols -E-cigarette smoking increased the ROS-producing enzyme NOX-2 (*p* < 0.01).
Olfert MI [36]	Animal	Ultrasound cardiac function and arterial stiffness (AS) with pulse wave velocity (PWV) in chronic exposure to E-cig vapor, standard (3R4F reference) cigarette smoke, or filtered air in mice.	-AS increased 2.5- and 2.8-fold in the E-cig- and 3R4F-exposed mice, respectively, compared with the air-exposed control mice (*p* < 0.05). -3R4F exposure altered cardiac function by reducing fractional shortening and ejection fraction after 8 months (*p* < 0.05). A similar, although not statistically significant, tendency was also observed with E-cig exposure (*p* < 0.10).
Rao P [37]	Animal	FMD in mice exposed to aerosol from e-liquids with and without nicotine, JUUL pods (Virginia Tobacco, Mango, and menthol), and an IQOS heated tobacco product; Marlboro Red cigarette smoke and clean air as controls.	-FMD was impaired by aerosol from previous generation e-cig (pre-exposure 9.8 ± 2.9% vs. post-exposure 5.4 ± 1.4%, *p* = 0.006), new-generation e-cig (11.2 ± 2.2% vs. 6.1 ± 2.3%, *p* = 0.0002), JUUL Virginia Tobacco (10.9 ± 3.5% vs. 5.6 ± 2.9%, *p* = 0.0001), JUUL Mango (10.5 ± 2.9% vs. 5.3 ± 2.7%, *p* = 0.0009), and JUUL Menthol (11.9 ± 3.4% vs. 6.4 ± 3.7%, *p* = 0.001), IQOS (11.2 ± 2.2% vs. 5.2 ± 3.2%, *p* = 0.0009), and Marlboro Red cigarette smoke (9.0 ± 3.3% vs. 3.2 ± 2.3%, *p* = 0.002) vs. no significant impairment of FMD was seen in the air group (7.8 ± 2.3% vs. 7.9 ± 4.3%, *p* = 0.98).
Carnevale R [38]	Human	Markers of oxidative stress, nitric oxide bioavailability, and vitamin E levels; flow-mediated dilation (FMD) measured in 40 healthy subjects (20 smokers and 20 non-smokers).	-In both e-cigarettes and traditional cigarettes significant increase in the levels of soluble NOX2-derived peptide and 8-iso-prostaglandin F2α and a significant decrease in nitric oxide bioavailability, vitamin E levels, and FMD. -Effects of e-cigarettes vs. traditional cigarettes on vitamin E levels (*p* = 0.413) and FMD (*p* = 0.311) were not statistically different. -E-cigarettes showed a lower impact than traditional cigarettes on levels of soluble NOX2-derived peptide (*p* = 0.001), 8-iso-prostaglandin F2α (*p* = 0.046), and nitric oxide bioavailability (*p* = 0.001).
Youn JY [39]	Human	Circulating nitrite levels in three different cohorts of young adults (n = 33, 21–25 years old): e-cigarette users (n = 13), tobacco cigarette smokers (n = 11), and nonusers (n = 9).	-Circulating nitrite levels were significantly lower in young adult e-cigarette users compared to those of nonusers (7.25 ± 0.45 vs. 11.06 ± 1.80, *p* < 0.05).
Antoniewicz L [40]	Human	Endothelial progenitor cells (EPCs) and microvesicles (MVs) in 16 healthy young volunteers randomized into two groups, either exposed or not to the inhalation of e-cigarette vapor (ECV).	-EPC levels in blood were significantly increased at 1 h and 4 h following exposure to ECV (*p* = 0.003 and *p* = 0.036, respectively) and returned to baseline values after 24 h. -No statistical differences in MV levels between the groups with the exception of CD62E positive MVs (*p* < 0.038).
Caporale A [41]	Human	Markers of endothelial function evaluated through magnetic resonance (MRI) in 31 non-smokers after the inhalation of aerosol from nicotine-free e-cigarettes.	-Resistivity index was higher (0.03 of 1.30 [2.3%]; *p* < 0.05), luminal flow-mediated dilation (−3.2% of 9.4% [−34%]; *p* < 0.001), along with reduced peak velocity (−9.9 of 56.6 cm/s [−17.5%]; *p* < 0.001), hyperemic index (−3.9 of 15.1 cm/s^2^ [−25.8%]; *p* < 0.001), and delayed time to peak (2.1 of 7.1 s [29.6%]; *p* = 0.005); baseline SvO_2_ was lower (−13 of 65%HbO_2_ [−20%]; *p* <0.001) and overshoot higher (10 of 19%HbO_2_ [52.6%]; *p* <0.001); and aortic pulse wave velocity marginally increased (0.19 of 6.05 m/s [3%]; *p* = 0.05). -No other parameters changed after aerosol inhalation.
Biondi-Zoccai G [42]	Human	Parameters of oxidative stress, antioxidant reserve, platelet function, FMD, and blood pressure in 20 traditional smokers, with allocation to different cycles of heat-not-burn cigarettes (HNBC), electronic vaping cigarettes (EVC), and traditional combustion cigarettes (TC).	-Single use of any product led to an adverse impact on oxidative stress, antioxidant reserve, platelet function, flow-mediated dilation, and blood pressure. -HNBC had less impact than EVC and TC on soluble Nox2-derived peptide (respectively, *p* = 0.004 and 0.001), 8-iso-prostaglandin F2α- III (*p* = 0.004 and <0.001), and vitamin E (*p* = 0.018 and 0.044). -HNBC and EVC were equally less impactful than TCs on flow-mediated dilation (*p* = 0.872 for HNBC versus EVC), H_2_O_2_ (*p* = 0.522), H_2_O_2_ breakdown activity (*p* = 0.091), soluble CD 40 ligand (*p* = 0.849), and soluble *p*-selectin (*p* = 0.821). -The effect of HNBC and, to a lesser extent, EVC, on blood pressure was less evident than that of TC, whereas HNBC appeared more satisfying than EVC (all *p* < 0.05).
Loffredo L [43]	Human	Parameters of oxidative stress and endothelial and platelet function in 78 children (2–18 years) divided into three groups: HNBC passive smokers (n = 26), traditional tobacco (TT) cigarette exposed (n = 26), and control (CNT) group (n = 26, unexposed).	-Significant increased serum sNOX2-dp (25.96 ± 5.26 TT, 24.87 ± 7.64 HNBC vs. 17.65 ± 7.92), isoprostanes (176.43 ± 43.75 TT, 178.5 ± 36.26 HNBC vs. 142.50 ± 20.89), H_2_O_2_ (32.35 ± 7.61 TT, 29.04 ± 6.13 HNBC vs. 23.19 ± 5.41), and sP-selectin (6.77 ± 1.92 TT, 6.33 ± 1.20 HNBC vs. 5.10 ± 1.74) in children exposed to the passive smoking of both HNBC and TT versus controls. -Exposed children showed a reduced brachial FMD (5.78 ± 2.92 TT, 5.51 ± 3.0 HNBC vs. 7.93 ± 2.30, *p* < 0.01) and serum NO bioavailability (49.92 ± 9.01 TT, 48.12 ± 11.15 HNBC vs. 60.69 ± 11.44 *p* < 0.001).
Loffredo L [4]	Human	An observational study assessing endothelial function, oxidative stress, and platelet activation in chronic smokers of traditional tobacco and users of heated tobacco products.	-Compared to non-smokers, the chronic smokers of TT and HNBC had lower brachial FMD [7.1 (2.8–11.5), 1.6 (0–3.9), and 3.3 (2.4–6.0)], nitric oxide (NO) bioavailability [41 (38–49), 10 (9–13), and 10 (8–13) (µM)], sNox2-dp (19 (15–23), 46 (41–50), and 40 (34–41) pg/mL), H_2_O_2_ [8.8 (7.2–11.9), 33.5 (19.5–52.7), and 26.7 (21.9–33.8) μM)], sCD40L [1.6 (1.1–2.1), 3.2 (2.5–4.4), and 3.0 (2.5–3.3) ng/mL], sP-selectin [3.0 (2.0–3.9), 9.2 (6.7–12.0), and 8.1 (5.5–9.2) ng/mL], and platelet aggregation [62 (58–70), 80 (77–80), and 76 (70–80)%].
Fetterman JL [44]		Arterial stiffness including carotid–femoral pulse wave velocity, augmentation index, carotid–radial pulse wave velocity, and central blood pressures in individuals without known cardiovascular disease or cardiovascular disease risk factors who were non-smokers (n = 94), users of combustible cigarettes (n = 285), users of e-cigarettes (n = 36), or dual users (n = 52).	-Combustible cigarette smokers had a higher augmentation index compared with nonusers (129.8 ± 1.5 versus 118.8 ± 2.7, *p* = 0.003). -The augmentation index was similar between combustible cigarette smokers compared with sole e-cigarette users (129.8 ± 1.5 versus 126.2 ± 5.9, *p* = 1.0) and dual users (129.8 ± 1.5 versus 134.9 ± 4.0, *p* = 1.0).
Boakye [45]	Human	FMD and reactive hyperemia index (RHI), high-sensitivity C-reactive protein, interleukin-6, fibrinogen, *p*-selectin, and myeloperoxidase in 46 participants (23 exclusive e-cigarette users; 23 nonusers).	- FMD was slightly lower among e-cigarette users (6.32%) compared to nonusers (6.53%); however, no statistically significant difference. -Levels of inflammatory markers were generally high but did not differ between e-cigarette users and nonusers.
Hamptonstall [46]	Human	FMD in healthy young people to compare the effects of acute and chronic tobacco cigarette (TC) smoking and electronic cigarette (EC): 47 non-smokers (NS), 49 chronic EC vapers, and 40 chronic TC smokers at baseline.	-Baseline FMD was not different among the groups (NS, 7.7 ± 4.5 vs. EC:6.6 ± 3.6 vs. TC, 7.9 ± 3.7%∆, *p* = 0.35), even when compared by group and sex. -Acute TC smoking versus control impaired FMD (FMD pre-/postsmoking, −2.52 ± 0.92 vs. 0.65 ± 0.93%∆, *p* = 0.02). -Acute EC vaping did not impair FMD.
Ikonomidis [48]	Human	Effects of heat-not-burn cigarette (HNBC) and tobacco cigarette (Tcig), on myocardial, coronary, and arterial function; oxidative stress; and platelet activation in 75 smokers.	-Acute HNBC smoking caused a smaller increase in PWV than Tcig (change 1.1 vs. 0.54 m/s, *p* < 0.05) without change in CO and biomarkers in contrast to Tcig. -Compared to Tcig, switching to HNBC for 1 month improved CO, FMD, CFR, TAC, GLS, GWW, MDA, and TxB2 (differences 10.42 ppm, 4.3%, 0.98, 1.8 mL/mmHg, 2.35%, 19.72 mmHg%, 0.38 nmol/L, and 45 pg/mL, respectively, *p* < 0.05).

## 6. Effect of Smoking on Platelets and Oxidative Stress

Platelet activation is another mechanism by which smoking can promote the development of atherosclerotic disease (Figure 1). The initial mechanism in thrombus formation is represented by the interaction of platelets with activated endothelial cells and with subendothelial matrix proteins, such as Von Willebrand factor (vWF), collagen, fibronectin exposed after tissue damage, and the consequent platelet activation [50]. Upon activation, platelets secrete more than 300 active substances from their intracellular granules, including p-selectin and adenosine diphosphate (ADP), and produce and secrete eicosanoids, such as thromboxane A2 and isoprostane F2, which amplify platelet activation and promote platelet aggregation [51].

ROS are produced in conditions of vascular damage by endothelial cells, leukocytes, muscle cells, and fibroblasts and can contribute to platelet activation [52]. However, several studies have shown that activated platelets are themselves able to produce ROS such as superoxide radical anion (O_2_^•−^), hydroxyl radical (^•^OH), and hydrogen peroxide (H_2_O_2_) [53,54,55]. Several enzymatic systems are involved in ROS production, including NADPH oxidase, cyclooxygenase (COX), eNOS, xanthine oxidase, and mitochondrial respiration [56]. Recent studies have shown that among the various mechanisms underlying platelet ROS production, NADPH oxidase 2 (NOX2) plays a central role. Indeed, NOX2 can influence platelet activation through many mechanisms, including the conversion of O_2_^•−^ to H_2_O_2_, a more stable and pro-aggregating metabolite [57]; the inhibition of the anti-aggregating activity of NO; and the transformation of arachidonic acid into F2-isoprostanes [58]. On the contrary, the inhibition of NOX2 activity has been shown to cause an alteration in the production of O_2_^•−^ and H_2_O_2_ and a reduction in calcium mobilization, resulting in reduced platelet aggregation [58].

The association between cigarette smoking and increased platelet activation has been demonstrated in several studies [19,59]. However, smoking from new-generation products (electronic cigarettes and HNBC) also seems to favor platelet activation and aggregation.

In vitro studies have shown that platelets from healthy donors exposed to e-cigarette smoke extracts displayed increased activity and aggregation, increasing platelet adhesion markers [60]. In vivo studies in mouse models have also observed a pro-thrombotic effect of e-cigarette smoke. Qasim et al. [61] observed a clear reduction in bleeding time in mice exposed to e-cigarette smoke compared to mice exposed to clean air; the exposure to e-cigarette smoke was associated with platelet hyperactivity, the increased expression of p-selectin on the cell surface, and reduced sensitivity to the inhibitory action of prostaglandins [61]. Ramirez et al. evaluated the effects of a specific type of electronic cigarette on platelet function. Specifically, platelets from e-cigarette-exposed mice showed increased cellular activity and aggregation relative to clean air-exposed control mice [62]. More recently, Snoderly et al. [63] demonstrated that in a murine model, short-term e-cigarette aerosol exposure significantly elevated neutrophil–platelet aggregates that are key drivers to the development or exacerbation of thrombo-inflammation [64].

Recent clinical studies have confirmed these data. A single-blind crossover study compared the effect of electronic cigarettes versus traditional cigarettes on platelet function in smokers and non-smokers [65]. Both traditional and e-cigarette smoking had a short-term impact on platelet activation, measured by soluble (s)CD40L and soluble (s)P-selectin, with a smaller effect in non-smokers [65]. The same authors compared the effect of HNBC smoking to e-cigarettes and traditional cigarettes, demonstrating an association of all three types of smoking with increased platelet activity [42]. However, the use of HNBC showed a smaller impact, compared to e-cigarettes and traditional cigarettes, on the production of 8-iso-PGF2α, an isoprostane with several biological functions including the propagation of platelet activation [66]. Furthermore, the concentrations of platelet biomarkers, such as sCD40L and sP-selectin, were less altered after the use of HNBC compared to traditional smoking [42].

In another recent study, Lyytinen et al. evaluated, in healthy subjects, the thrombotic effect induced by brief electronic cigarette inhalation on flow-dependent thrombus formation measured by the Total Thrombus Formation Analysis System (T-TAS). They observed an increased platelet thrombus formation and fibrin-rich thrombus formation 15 min after exposure [67]. The same authors then evaluated the thrombotic effect induced by HNBC smoking by using T-TAS, observing, in this case, an immediate increase in the formation of platelet thrombi 5 min after exposure [68].

Passive smoking from HNBC has also been shown to induce platelet activation, in a pediatric population, in a similar manner to that induced by traditional cigarette smoking [43]. Particular attention has also been paid to the effect of “thirdhand” smoking, which refers to the persistent residual contamination that adheres to surfaces and reemits into the air. In a recent study, using a mouse thirdhand e-cigarette exposure protocol, it has been demonstrated that chronic exposure (4 months) increased platelet activation and aggregation in vitro and shortened tail bleeding and occlusion times in vivo [69]. These results highlight the effect of this type of exposure on hemostasis and therefore, a potentially increased risk of occlusive cardiovascular disease [69].

## 7. Cardiovascular Effects of Smoking on Children

In children, exposure to cigarette smoke, whether active or passive, is particularly concerning due to the long-term health implications, especially concerning the cardiovascular system [5,70]. The harm caused by cigarette smoke, particularly in terms of passive exposure, is widely documented in the literature regarding children’s respiratory pathways [5,71,72]. Smoking acts as an inflammatory trigger that significantly contributes to the onset of asthma, rhinitis, allergies, and obstructive sleep apnea syndrome. These respiratory conditions are characterized by an increased cardiovascular risk in adults, likely due to the persistence of an inflammatory state [73]. However, the initial damage occurs very early. Recent studies suggest that exposure to secondhand smoke in early childhood, even in utero, can cause persistent changes in lipoproteins. Ayer et al. documented that prenatal exposure to secondhand tobacco smoke was associated with lower levels of HDL-C (high-density lipoproteins) in children aged 8 years [74]. This increased risk seems to extend beyond cholesterol levels. It has been observed that children exposed to secondhand smoke have a significantly higher incidence of metabolic syndrome, at 19.6%, compared to 5.6% in non-exposed children. Exposure to secondhand tobacco smoke, as evidenced by cotinine concentrations, has been associated with impaired endothelial function in a dose-dependent manner in preadolescent children (8–11 years) [75]. Numerous studies have also analyzed the deleterious cardiovascular effects of e-cigarettes, traditional cigarettes, and heated tobacco products in adolescents [76]. Nicotine consumption is associated with increased blood pressure and heart rate [76]. The use of electronic cigarettes increases airway resistance, makes inhalation more difficult, and contributes to the production of highly reactive free radicals, causing an increase in oxidative stress, which in turn is linked to mitochondrial dysfunction and reduced nitric oxide bioavailability [77]. The consequences of these pathological mechanisms include endothelial dysfunction and cardiotoxicity, understood as structural and functional cardiac damage [77]. Cardiotoxicity can be irreversible in cases of myocardial cell necrosis or apoptosis, or reversible, as in the case of short-term nicotine product use (or even long-term smoking in heavy smokers) [78]. Clinical manifestations include paroxysmal or permanent arrhythmias, systolic and/or diastolic dysfunction, and heart failure [78]. Therefore, exposure to cigarette smoke, whether from traditional tobacco or e-cigarettes, through mechanisms related to increased oxidative stress and inflammation, contributes to triggering an inflammatory state that, if sustained over time, may increase the risk of cardiovascular complications in adulthood.

## 8. Cardiovascular Effects of Smoking in Adults

**Figure 4:**
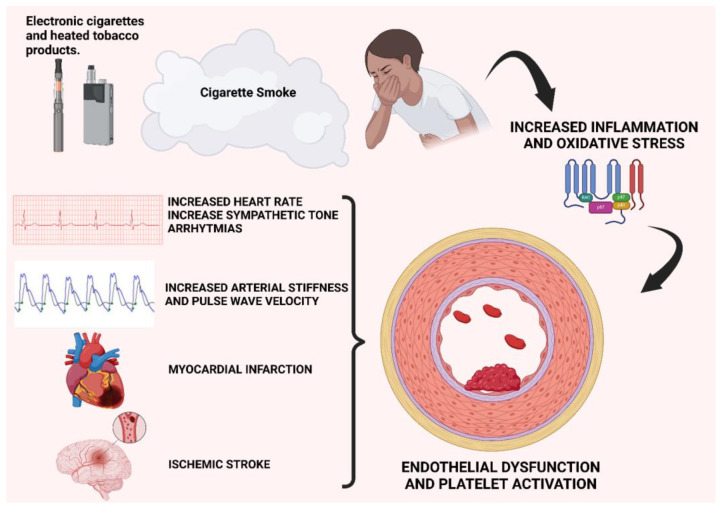
Cardiovascular effects of e-cigarette and HNBC smoke. Both the active and passive inhalation of e-cigarette and heat-not-burn cigarette (HNBC) smoke lead to increased oxidative stress and inflammatory states, resulting in platelet activation and endothelial dysfunction—mechanisms that underlie atherosclerotic disease. An elevated cardiovascular risk, including increased susceptibility to coronary artery disease and stroke, has been demonstrated both in adult smokers of new-generation products and in children exposed to secondhand smoke. In particular, in children, exposure to secondhand e-cigarette and HNBC smoke appears to have both a direct cardiovascular effect—by increasing oxidative stress, endothelial dysfunction, and platelet activation—and an indirect effect through chronic inflammation associated with asthma, which is often a consequence of smoke exposure in children.

Over the past decades, a clear association has been established between tobacco smoking and thrombotic [79,80,81,82] and cardiovascular events [83]. Electronic cigarettes (e-cigarettes) and HNBC were initially developed to support the cessation of tobacco smoking, offering a less harmful alternative to traditional cigarettes. However, the number of consumers of these new-generation tobacco products has significantly increased, including among non-smokers, due to the misconception that these products are not harmful. While they exhibit lower toxicity compared to traditional cigarettes, these products still exert effects on the cardiovascular system (Figure 4).

The available data on the cardiovascular effects of e-cigarettes and HNBC primarily come from short-term studies; data on long-term effects are lacking. An increase in arterial stiffness and pulse wave velocity (PWV) has been observed in the users of both e-cigarettes and HNBC [68,84,85,86]. Some studies also indicate that using nicotine-containing e-cigarettes can lead to acute effects on hemodynamic parameters, such as increased heart rate and blood pressure [84,85,87,88,89]. E-cigarette smoking appears to increase sympathetic tone, which is associated with a higher cardiovascular risk, and induce changes in electrocardiographic parameters of ventricular repolarization, which are linked to an elevated risk of sudden cardiac death, albeit to a lesser extent than traditional cigarette smoking [90,91,92]. According to another recent study, HNBC smoking has shown an acute impact on systolic and diastolic myocardial function comparable to that induced by traditional cigarette smoking [93]. In contrast, other studies have demonstrated benefits in transitioning from traditional cigarettes to e-cigarettes, with reductions in blood pressure and heart rate, and improvements in vascular function parameters [94,95,96,97]. Additional studies have explored the correlation between e-cigarette use and the incidence of myocardial infarction, finding an increased risk among the users of these products, even after adjusting for other cardiovascular risk factors [98,99]. A recent cross-sectional study highlighted lower overall health scores and a higher incidence of chest pain, palpitations, arrhythmias, and coronary artery disease among e-cigarette users compared to non-smokers [100]. However, some studies present opposing data. A large cohort study [101] based on self-reported cardiovascular events found no correlation between e-cigarette use and cardiovascular diseases (coronary artery disease, myocardial infarction, and stroke) among individuals who had never smoked before, while the “dual use” of traditional and e-cigarettes was associated with a 36% higher risk of cardiovascular events compared to exclusive traditional cigarette smokers. Similarly, in another study by Berlowitz et al., no difference in the risk of cardiovascular events was found between e-cigarette users and non-smokers, whereas dual users had an increased cardiovascular risk [102].

Recently, a study published by Kang [103] showed that, among patients undergoing percutaneous angioplasty, those who switched from traditional cigarettes to e-cigarettes or quit smoking altogether experienced fewer major cardiovascular events. The retrospective nature of the study limits definitive conclusions; future prospective studies will be needed to confirm these findings, which could have positive implications in this field.

The significant discrepancies in the results of these studies are mainly due to differences in study populations, exposure types, and the variety of products and models. Moreover, data on the long-term effects of new-generation tobacco products are lacking due to their recent introduction to the market.

The pro-thrombotic effect induced by tobacco smoking also extends to the cerebrovascular system. Combustible cigarette smoking increases the risk of neurological conditions, including stroke and vascular dementia. Tobacco smoking can damage vascular endothelial function and the blood–brain barrier (BBB), with long-term consequences for the brain [104,105]. Oxidative stress is believed to play a central role in the pathogenesis of vascular damage in these patients [106]. E-cigarettes and HNBC are not exempt from cerebrovascular effects. An interesting finding from a recent cross-sectional survey study of over 160,000 subjects is that the use of e-cigarettes alone was not associated with an increased risk of stroke; however, if young adults had a history of previous traditional cigarette smoking or were dual users, the risk of stroke significantly increased, even compared to exclusive traditional cigarette smokers [107]. Pre-clinical studies have observed that nicotine, present in both traditional cigarettes and new-generation products, can cause oxidative damage similar to that seen with tobacco smoking, resulting in the disruption of BBB integrity [108]. Additionally, exposure to e-cigarette smoke has been shown to be associated with exacerbated cerebral ischemic damage and secondary brain injury in murine models [108,109,110]. Another effect of nicotine is the reduction in cerebral glucose utilization: prolonged exposure to nicotine, whether from traditional or e-cigarettes, induces an increase in the expression of GLUT1 in the brain, leading to a reduction in glycolysis, which causes a condition of glucose deprivation that increases the risk of stroke and worsens ischemic damage [111]. It has also been observed that cigarette smoking is associated with an increased risk of developing glucose intolerance and type 2 diabetes mellitus, which represents another important cerebrovascular risk factor [112]. Recent studies have observed that BBB endothelial cells, in response to hyperglycemia and/or ischemic damage, increase the production of ROS and inflammation modulators, suggesting a common pathogenic mechanism of the BBB in these conditions [113]. In this regard, the use of metformin before and after ischemic damage has been associated with a reduction in oxidative stress and inflammatory response [108].

Even the smoking of new-generation products could increase the atherosclerotic and thrombotic risk in adults through the mechanisms of inflammation, platelet activation, and endothelial dysfunction.

## 9. Conclusions

While heated tobacco products and electronic cigarettes contain fewer toxic substances compared to traditional tobacco, they may not be without cardiovascular health risks. These products may have pro-atherosclerotic and pro-thrombotic effects by increasing oxidative stress, causing endothelial dysfunction, and promoting platelet activation (Figure 1, Figure 2 and Figure 3).

Given this evidence, it is clear that the risk of atherosclerosis that could be associated with electronic cigarettes and heated tobacco cannot be overlooked. These products are often used by smokers who are unable to quit; however, it is essential to promote stricter regulations and raise awareness of their potential health risks. Although heated tobacco products may reduce certain toxic effects compared to cigarette smoking, no tobacco product is completely free of health risks. Efforts to prevent and reduce the use of electronic cigarettes and heated tobacco among young people are especially important given the growing popularity of these products among teenagers. Targeted awareness campaigns and educational interventions can help inform the public about the health risks associated with these tobacco alternatives and encourage healthier behaviors. Steven Dodd et al., through their systematic review, summarize the effectiveness of the current health interventions guided by “peer education”, where adolescents teach their peers about the risks and harmful effects of smoking. This strategy seems to be promising for encouraging the prevention and cessation of smoking among young people [114].

In conclusion, the risk of atherosclerosis linked to electronic cigarettes and heated tobacco could represent a significant threat to cardiovascular health, especially in children. It is essential to continue conducting in-depth research to fully understand the effecs of these products on the cardiovascular system and to implement concrete measures to protect public health.

## Supplementary Materials

The following supporting information can be downloaded at https://www.mdpi.com/article/10.3390/antiox13111395/s1. Table S1: Studies included in the systematic review evaluating the chronic effects of exposure to heated tobacco or electronic cigarette smoke on endothelial dysfunction in the brachial artery.
