# Supplementary material for: The Hidden Dangers: E-Cigarettes, Heated Tobacco, and Their Impact on Oxidative Stress and Atherosclerosis—A Systematic Review and Narrative Synthesis of the Evidence
Source: Antioxidants (Basel). 2024 Nov 15;13(11):1395. doi: 10.3390/antiox13111395 (PMC11591068; doi:10.3390/antiox13111395)
Supplement: Supplementary file 1 [file antioxidants-13-01395-s001.zip › antioxidants-3295308-supplementary.pdf]

Supplementary data:

Eligibility criteria

Types of studies: studies that assessed the effect of electronic cigarettes or heated tobacco cigarettes on FMD in subjects exposed passively or actively to the smoking. No language, publication date, or publication status restrictions were imposed.

Information sources

The studies were identified by searching electronic databases. search was applied to Pubmed, ISI Web of Science, SCOPUS and Cochrane database. The last search was run on October 21, 2024. Reference lists of all studies included in the present meta-analysis were screened for potential additional eligible studies.

Search

Two investigators independently searched in the electronic databases combining the following text terms and MeSH terms: Entry Terms:

- Electronic Nicotine Delivery System, Electronic Cigarettes, Electronic Cigarette, Cigarette, Electronic, Cigarettes, Electronic, E-Cigs, E Cigs, E-Cigarettes, E Cigarettes, E-Cigarette, E Cigarette, E-Cig, E Cig, heated tobacco products, heated tobacco, IQOS, HBNC. We limited our search to human studies.

Study selection

Two authors independently reviewed the titles and abstracts generated by the search. Studies were excluded if the title and/or abstract showed that the papers did not meet the selection criteria of our systematic review. Case reports, editorials, commentaries, letters, review articles, guidelines were also excluded from the analysis. We defined the following exclusion criteria: studies that included only acute effect of the cigarettes, studies that switched from different types of tobacco, studies unrelated to our topic. A flowchart of the selection studies is reported below.

Main analysis

Our primary outcome was to compare the effect of passive or active e-cigarettes or heated tobacco products on endothelial dysfunction evaluated by brachial FMD. This systematic review was conducted and reported according to the PRISMA (Preferred Reporting Items for Systematic Reviews and Meta-Analysis)[1].

Statistical analysis

We allocated the results of each study reporting mean and standard deviation of brachial FMD.

The software Comprehensive Meta Analysis (version 2.2.064, USA, 2011) and R (version 3.1.2, Vienna, 2014) supported the analysis.

Six studies[2-7] (S-Table 1)evaluated the effect of E-cigarettes or Heated tobacco cigarettes on FMD.

Table S1

Study	Type of smoking	Type of studied population	Exposure	Age	Gender
Loffredo	Heated Tobacco and Traditional Tobacco	Children	Passive	9 ±3 years (not exposed), 9 ±3 years (traditional tobacco), 10 ±3 years (heated tobacco)	26 males and 52 females
Loffredo	Heated Tobacco and Traditional Tobacco	Adults	Active	28 (23–33) years (nonsmokers), 27 (24– 30) years (traditional tobacco), 33 (28–44) years (heated tobacco)	27 males and 33 females
Mohammadi	E-Cigarettes, Traditional Tobacco and nonsmokers	Adults	Active	29±4,6 years (e-cigarettes), 34±8 years (traditional tobacco), 28±4 years (nonsmokers)	73 males and 47 females
Fetterman	E-Cigarettes, Traditional Tobacco, Dual Users and nonsmokers	Adults	Active	29±6 years (nonsmokers), 32±7 years (traditional tobacco), 29±6 (e-cigarettes), 33±7 (dual users)	294 males and 173 females
Boakye	E-Cigarettes and nonsmokers	Adults	Active	25,6 ± 4,0 years (nonsmokers), 23,0 ± 3,7 years (e-cigarettes)	36 males and 10 females
Haptonstall	E-Cigarettes, Traditional Tobacco and nonsmokers	Adults	Active	26,3 ± 5,20 years (nonsmokers), 27,4 ± 5,45 years (e-cigarettes), 27,1 ± 5,51 years (traditional tobacco)	84 males and 52 females

References:

[1] Page MJ, McKenzie JE, Bossuyt PM, Boutron I, Hoffmann TC, Mulrow CD, et al. The PRISMA 2020 statement: an updated guideline for reporting systematic reviews. BMJ. 2021;372:n71.
[2] Loffredo L, Carnevale R, Pannunzio A, Cinicola BL, Palumbo IM, Bartimoccia S, et al. Impact of heat-not-burn cigarette passive smoking on children's oxidative stress, endothelial and platelet function. Environ Pollut. 2024;345:123304.
[3] Loffredo L, Carnevale R, Battaglia S, Marti R, Pizzolo S, Bartimoccia S, et al. Impact of chronic use of heat-not-burn cigarettes on oxidative stress, endothelial dysfunction and platelet activation: the SUR-VAPES Chronic Study. Thorax. 2021;76:618-20.
[4] Mohammadi L, Han DD, Xu F, Huang A, Derakhshandeh R, Rao P, et al. Chronic E-Cigarette Use Impairs Endothelial Function on the Physiological and Cellular Levels. Arterioscler Thromb Vasc Biol. 2022;42:1333-50.
[5] Fetterman JL, Keith RJ, Palmisano JN, McGlasson KL, Weisbrod RM, Majid S, et al. Alterations in Vascular Function Associated With the Use of Combustible and Electronic Cigarettes. J Am Heart Assoc. 2020;9:e014570.
[6] Boakye E, Uddin SMI, Osuji N, Meinert J, Obisesan OH, Mirbolouk M, et al. Examining the association of habitual e-cigarette use with inflammation and endothelial dysfunction in young adults: The VAPORS-Endothelial function study. Tob Induc Dis. 2023;21:75.
[7] Haptonstall KP, Choroomi Y, Moheimani R, Nguyen K, Tran E, Lakhani K, et al. Differential effects of tobacco cigarettes and electronic cigarettes on endothelial function in healthy young people. Am J Physiol Heart Circ Physiol. 2020;319:H547-H56.
